# Annotations

**Published:** 1911-04-22

**Authors:** 


### Annotations.

### The Drawbacks of Hygienic Methods.

The Italians would seem to be taking to hygienic methods with avidity. The Mayor of Rome has recently issued a notice in which, among other things, it is forbidden for buyers to finger bread and other products of a similar nature in order to test their freshness or degree of baking. The idea is an excellent one, but the question naturally arises as to how one is to get the desired information without making use of the antiquated method of touching the articles. For example, a loaf may be beautifully cooked on top, and yet the bottom of it be burnt to a cinder! To trust to the word of the tradesman presents certain risks in Italy as elsewhere. There is but one other solution of the difficulty, and that is to oblige the customer to wash his hands at the baker's and under the latter's supervision. Possibly this might be a new experience for some shop-goers!

### Ætiological Fads.

The theory that appendicitis is caused, in some occult way, by the eating of adulterated bread has now attained the doubtful honour of Parliamentary comment, and the result will probably be that an entirely unsupported hypothesis will obtain an entirely undeserved popularity until it is replaced in turn by some equally ingenious suggestion. The ætiology of appendicitis has offered a wide scope for the exercise of the imaginative faculty among medical men, and the number of those who have availed themselves of the opportunity to formulate new theories of causation is as large—well, as the number of complications of the disease. There are, we believe, no fewer than forty such theories which have at one time or other been put forward to account for the increase in the frequency of the condition, and it is not unfair to say that not one of them has as yet been proven. The soap theory, the "iron fragment" theory (which may justifiably be called the original bread theory), the aërated water theory, and a host of others have been discussed with an energy and a seriousness worthy of a better cause, and there is even now in process of manufacture, we understand, an elaborate report on an brand new hypothesis put forward by an Italian investigator. Dr. Charles Mercier did good service to the medical profession in particular, and to science in general, when he suggested that in the decay of wig-wearing may possibly be found an explanation of the increased frequency of appendicitis. It is only by showing the facility with which the reduction to the absurd may be attained that such quodlibetical discussion can be killed. Ætiological fads manufactured in the laboratory stand in need of some sarcastic criticism.

### The Troubles in Russian Universities.

The last circulars of the Russian Minister of Public Instruction and of the Ministerial Council directed towards the eradication of the last traces of university autonomy have provoked much disturbance and the cessation of study in the greater part of the universities and superior schools in Russia. The draconian measures taken by the Government (during a single month over a thousand students have been expelled and forbidden to reside in University towns, several hundreds being arrested, etc.), have only aggravated the situation, and now a total absence of instruction is the order of the day in the Universities of St. Petersburg and Moscow. The crisis is spreading to the provinces. At Moscow the posts of rector and vice-rector have been declared vacant by order of the Minister. This untenable and shameful position in which the University finds itself placed has provoked the resignation of a large number of professors and fellows. Every day sees more resignations, so that the oldest and largest University of Russia, with more than ten thousand students, is threatened in its very existence. The Faculty of Science is the most attacked. That of medicine is little better off. In addition to Professor Minakow, who has been dismissed, many well-known members of the staff have sent in their resignations.

### Boyhood and Physical Training.

Austria has decided to fall into line with Germany in providing for the efficient physical training of her schoolboys. As the result of a prolonged Departmental Commission, appointed by the Education Office at Vienna, a concise scheme has been put forward, which provides for the formation of boys clubs and gymnastic classes in all public schools, together with compulsory instruction of drilling and in military hygiene. The course of instruction will also include musketry and target shooting. This arrangement will doubtless be considered an abomination by those who see in every attempt to teach the modern boy the elements of discipline, self-reliance, co-operation, and communal control an endeavour to inculcate the pernicious principles of militarism and to encourage the manufacture of aggressive Chauvinists on a large scale among the juvenile population. But those who are with us in the opinion that there is a vast difference between the physical education of the young along lines which wide experience and extended trial have shown to be the best in dealing with the mass of raw material, and that it is imperative that such training should be started early in life, will find in the scheme an example which is at least worth consideration in this country. We have no wish to refer in detail to the interesting syllabus drawn up for the musketry course, but it is worth while pointing out that physical drill without some object in view that is apparent to the drilled only half fulfils expectations. The average boy, in this country or elsewhere, will find in this course of musketry drill an interest which staves and dumb-bells cannot replace, and it is just as well that instructors should utilise this interest to add to the benefit which such courses may bring by teaching judgment of range and distance, observation, and accuracy. Those who are acutely frightened at the bogey of militarism will be interested to find that as the result of the adoption of this scheme of physical training in schools, Austria hopes to reduce to two years the period of compulsory service in the Army which every Austrian has now to undergo.

